# Fetoplacental Vascular Endothelial Dysfunction as an Early Phenomenon in the Programming of Human Adult Diseases in Subjects Born from Gestational Diabetes Mellitus or Obesity in Pregnancy

**DOI:** 10.1155/2011/349286

**Published:** 2011-11-24

**Authors:** Andrea Leiva, Fabián Pardo, Marco A. Ramírez, Marcelo Farías, Paola Casanello, Luis Sobrevia

**Affiliations:** ^1^Cellular and Molecular Physiology Laboratory (CMPL) and Perinatology Research Laboratory (PRL), Division of Obstetrics and Gynaecology, School of Medicine, Faculty of Medicine, Pontificia Universidad Catolica de Chile, P.O. Box 114-D, Santiago, Chile; ^2^Biomedical Department, Faculty of Health Sciences, Universidad de Antofagasta, Antofagasta, Chile

## Abstract

Gestational diabetes mellitus (GDM) and obesity in pregnancy (OP) are pathological conditions associated with placenta vascular dysfunction coursing with metabolic changes at the fetoplacental microvascular and macrovascular endothelium. These alterations are seen as abnormal expression and activity of the cationic amino acid transporters and endothelial nitric oxide synthase isoform, that is, the “endothelial L-arginine/nitric oxide signalling pathway.” Several studies suggest that the endogenous nucleoside adenosine along with insulin, and potentially arginases, are factors involved in GDM-, but much less information regards their role in OP-associated placental vascular alterations. There is convincing evidence that GDM and OP prone placental endothelium to an “altered metabolic state” leading to fetal programming evidenced at birth, a phenomenon associated with future development of chronic diseases. In this paper it is suggested that this pathological state could be considered as a metabolic marker that could predict occurrence of diseases in adulthood, such as cardiovascular disease, obesity, diabetes mellitus (including gestational diabetes), and metabolic syndrome.

## 1. Introduction

**Figure 1:**
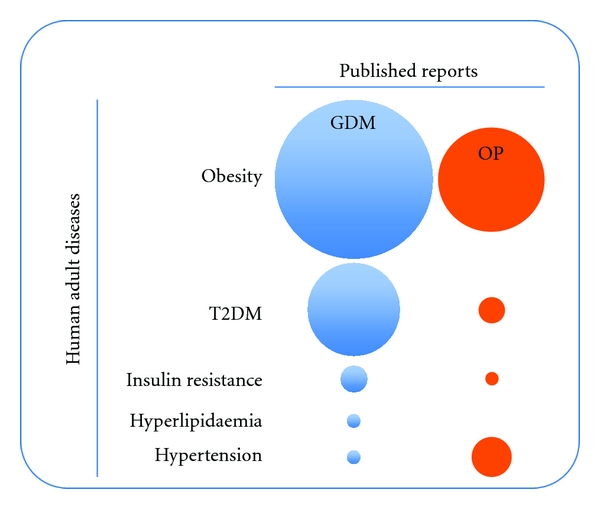
Comparison of published reports addressing a potential association of human adult diseases in subjects from pregnancies coursing with gestational diabetes mellitus or obesity in pregnancy. Gestational diabetes mellitus (GDM, column of light-blue circles) and obesity in pregnancy (OP, column of orange circles) are pathological conditions in human subjects. Different number of reports (*x*-axis, *Published reports*), in this cartoon represented as relative size of corresponding light-blue and orange circles, suggest that GDM and OP are differentially associated with increased incidence of human adult diseases (*y*-axis, *Human adult diseases*), such as obesity, type 2 diabetes mellitus (T2DM), insulin resistance, hyperlipidaemia, or hypertension. Data taken from [13, 77, 78, 81, 84, 92, 190, 195–212].

Pregnancy is a physiological state with a complex anatomical and functional interaction between mother and fetus [1]. When this interaction is not a success, the mother, the fetus, or both exhibit functional impairments. Complications of pregnancy are important causes of maternal mortality, where gestational diabetes mellitus (GDM) and obesity of the mother in pregnancy (OP) are major obstetric pathologies. Fetal-maternal interaction could result in metabolic disturbances leading, for example, to placental and endothelial dysfunction [2, 3]. Endothelial dysfunction is defined as an altered capacity of the endothelium to take up and metabolize the cationic amino acid L-arginine, the substrate for nitric oxide (NO) synthesis via NO synthases (NOS) [4, 5]. Interestingly, it is reported that GDM and OP are pathological conditions associated with altered L-arginine transport and NO synthesis (i.e., the “L-arginine/NO signalling pathway”), probably due to altered uptake and metabolism of adenosine [6, 7], an endogenous nucleoside acting as vasodilator in most vascular beds [8, 9]. These pathophysiological characteristics are considered key in the establishment of a “programmed state” of the developing fetus (i.e., “fetal programming”). This concept refers to the impact of abnormal intrauterine conditions on the development of diseases in adulthood and becomes a key mechanism associated with future development of chronic diseases including cardiovascular disease (CVD), diabetes mellitus, and metabolic syndrome (a concept globalizing clinical association of obesity, type II or non-insulin-dependent diabetes mellitus, hypertension, and dyslipidaemia) [10–12]. Interestingly, GDM is a condition that also increases the risk of obesity in children and adolescents [13], a phenomenon leading to high incidence of type 2 diabetes mellitus (T2DM) [14]. OP is also related to neonatal metabolic compromise, which is already apparent in the offspring at birth, characterized by reduced insulin sensitivity and higher concentrations of inflammatory markers [13]. Surprisingly, few studies have been reported regarding the potential association between GDM and OP as pathological conditions of the mother during pregnancy leading to diseases in the adulthood, the latter most likely programmed during the intrauterine life period (Figure 1). These concepts are discussed in this paper in terms of the fetus-placenta interaction and consequences of GDM and OP leading to fetal vascular disturbances. We also suggest that, based in the discussed observations, our attention should be certainly switched towards a better understanding of the gestational period as a key interventional target in the prevention of adult diseases at the state where fetal programming of adult diseases occurs.

## 2. Endothelial Dysfunction

**Figure 2:**
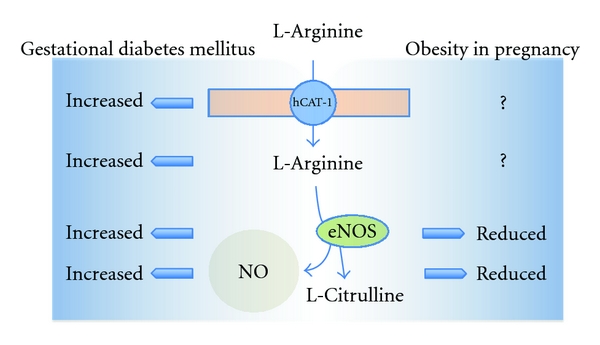
Endothelial L-arginine/NO signalling pathway in gestational diabetes mellitus and obesity in pregnancy. In human endothelial cells L-arginine is taken up via cationic amino acid transporters 1 (hCAT-1) accumulating this amino acid in the intracellular space. L-Arginine is then metabolized by the endothelial nitric oxide synthase (eNOS) into L-citrulline and nitric oxide (NO) as a co-product. Gestational diabetes mellitus is associated with higher expression and activity of hCAT-1 leading to supraphysiological accumulation of L-arginine. This phenomenon results in higher L-arginine metabolism by eNOS due to increased expression and activity of this enzyme leading to overproduction of NO. In endothelial cells from OP there is no information addressing whether this pathological condition alters L-arginine transport and intracellular accumulation, but reduces eNOS expression and activity leading to lower than physiological synthesis of NO.

Endothelial cells play a crucial role in the regulation of vascular tone through the release of vasoactive substances, including nitric oxide (NO) [4, 5, 15]. In pathological pregnancies, such as GDM [6, 16], intrauterine growth restriction (IUGR) [2], or preeclampsia [17], the synthesis and/or bioavailability of NO are altered leading to changes in blood flow of the human placenta which could result in limiting fetal growth and development [1, 3]. NO is a gas synthesized from the cationic, semiessential amino acid L-arginine in a metabolic reaction leading to equimolar formation of L-citrulline and NO (Figure 2) [5]. This reaction requires the activity of NO synthases (NOS), of which at least three isoforms have been identified, that is, neuronal NOS (nNOS or type I), inducible NOS (iNOS or type II), and endothelial NOS (eNOS or type III) [4, 5, 18]. The NO diffuses from the endothelium to the underlying layer of vascular smooth muscle cells leading to cyclic GMP (cGMP)-dependent vasodilatation [5]. In vessels without innervation, such as the placenta and the distal segment of the umbilical cord [1, 19], vascular tone is regulated by the synthesis and release of vasoconstrictors and vasodilators from the endothelium [3]. The reduced ability of this tissue to stimulate NO-mediated vasodilatation is referred to as endothelial dysfunction [20]. This phenomenon is strongly correlated with cardiovascular disease (CVD) risk factors [21] and with early states of chronic diseases such as hypertension [22], hypercholesterolemia [23], diabetes mellitus [24], hyperhomocysteinaemia [25], and chronic renal [26] and cardiac failure [27]. Interestingly, eNOS expression and activity is highly regulated in human fetoplacental microvascular and macrovascular endothelium, an effect that is differential in these two vascular beds; thus, endothelial dysfunction and perhaps increased risk of appearance of chronic diseases in adulthood will also depends on the type of fetal vascular bed that is altered in diseases of pregnancy [16].

**Figure 3:**
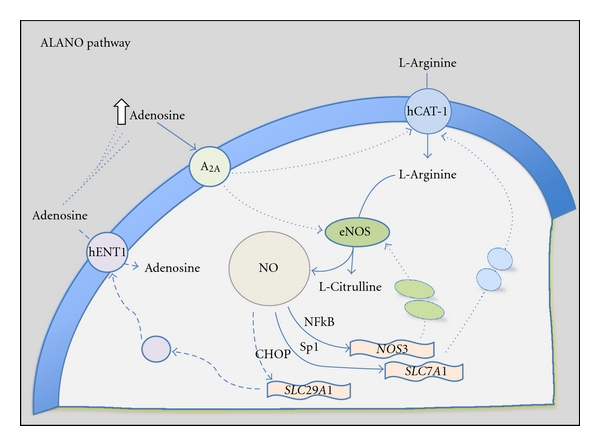
Adenosine/L-arginine/nitric oxide (ALANO) signalling pathway in gestational diabetes mellitus. Human umbilical vein (macrovasculature) and placental microvascular endothelial cells exhibit increased (solid light-blue arrows) L-arginine transport via the cationic amino acid transporters 1 (hCAT-1) but reduced (segmented light-blue arrows) adenosine uptake via the equilibrative nucleoside transporter 1 (hENT1). The latter phenomenon leads to accumulation (white up arrow) of adenosine in the extracellular space, which then stimulates A_2A_ adenosine receptors to activate (dotted light-blue arrows) maximal transport capacity of hCAT-1 and maximal metabolic capacity of endothelial nitric oxide synthase (eNOS) leading to supraphysiological levels of nitric oxide (NO) and L-citrulline. The gas NO activates hC/element-binding protein (CBP) homologous protein 10-C/EBP*α* transcription factor complex (CHOP) leading to repression of *SLC29A1 *gene expression resulting in reduced hENT1 protein synthesis and abundance at the plasma membrane. On the other hand, NO activates the transcription factor-specific protein 1 (Sp1) and nuclear factor *κ*B (NF*κ*B) leading to increase transcription of *SLC7A1* and *NOS3* genes, respectively. This phenomenon results in higher abundance of hCAT-1 and eNOS protein increasing L-arginine transport and NO synthesis. From data in [6, 16, 39, 48, 52, 59].

Activity of NOS may depend on the ability of endothelial cells to take up their specific substrate L-arginine via a variety of membrane transport systems [2, 28–30]. In human endothelial cells, L-arginine is taken up via membrane transport systems grouped as systems y^+^, y^+^L, b^0,+^, and B^0,+^ [31–33]. System y^+^ conforms a family of proteins known as cationic amino acid transporters (CATs) (hereafter referred as “CATs family”), with CAT-1, CAT-2A, CAT-2B, CAT-3, and CAT-4 isoforms [34] whose expression and activity, and the mechanisms modulating these phenomena, have been extensively described [30, 33–35], including in the human placenta [36–38]. Human fetoplacental endothelium takes up adenosine via equilibrative nucleoside transporters (ENTs) [6, 16, 39–41]. Four members of the ENT family of solute carriers (*SLC29A* genes) have been cloned from human tissues, that is, hENT1, hENT2, hENT3 and hENT4 [40, 41]. In primary cultures of human umbilical vein endothelial cells (HUVECs), adenosine transport is mainly (~80%) mediated by hENT1 with the remaining transport (~20%) being mediated by hENT2 [39, 42, 43]. Recent reports show that these proteins are also expressed in human placental microvascular endothelial cells (hPMECs); however, contribution of hENT1 and hENT2 to total adenosine transport in this cell type is similar compared with adenosine transport in HUVEC [17, 44]. hENT3 and hENT4 seem not to play a significant role in endothelium (see [16, 45–47]). Interestingly, adenosine has been suggested as a nucleoside increasing L-arginine/NO signalling pathway in HUVEC [39, 48], hPMEC [17, 49], rat cardiomyocytes in response to the ENTs inhibitor dipyridamole [41], and in skeletal microvascular endothelium in response to hypoxia [50]. This phenomenon has been referred to as endothelial “ALANO” signalling pathway (adenosine/L-arginine/nitric oxide) first characterized in HUVEC from GDM pregnancies [6, 16, 48]. The mechanism involves adenosine activation of A_2A_-adenosine receptors and increased expression of hCAT-1 and eNOS, via activation of key signalling molecules including mitogen-activated protein kinases of 42 and 44 kDa (p42/44^mapk^) and protein kinase C (PKC) [6, 7, 16, 39, 48]. Thus, a relationship between expression and activity of hCATs and hENTs in HUVEC from GDM has been established (Figure 3) [6, 16, 48, 51, 52].

## 3. Gestational Diabetes Mellitus

Gestational diabetes mellitus (GDM) is a syndrome characterized by glucose intolerance leading to maternal hyperglycaemia first recognized during pregnancy [53]. GDM is associated with abnormal foetal development and perinatal complications, such as macrosomia and neonatal hypoglycaemia [54]. Alterations associated with GDM result from a change in the amount of D-glucose available to the fetus due to alterations in the physiology of the placenta (e.g., increased D-glucose transplacental transport) or by hormone-induced dysfunction (e.g., altered insulin signalling), phenomena that could lead to abnormal growth of the fetus (macrosomia) and perinatal complications [16, 55, 56]. Clinical manifestations of GDM have been attributed mainly to the condition of hyperglycaemia, hyperlipidaemia, hyperinsulinemia, and fetoplacental endothelial dysfunction [54, 55]. Various organs show structural and functional alterations, including endothelial dysfunction of the micro- and macrocirculation in the fetoplacental circulation, in GDM [16, 57]. Increased NO synthesis has also been reported in human placental veins and arteries [58] and in primary cultures of HUVEC [7, 51, 59] isolated from pregnancies with GDM (Table 1). Thus, vascular dysfunction resulting from this syndrome may be a consequence of a functional dissociation between the synthesis of NO and/or its bioavailability to the vascular endothelium and smooth muscle in the human placenta circulation. Even when the GDM-associated endothelial dysfunction regards altered endothelial L-arginine/NO signalling pathway, most studies regarding the mechanisms behind these effects of GDM are not conclusive. However, it is conceivable that these alterations are the result of alterations in multiple, rather than single, metabolic mechanisms including sensitivity of the human fetal endothelium to vasoactive molecules such as adenosine [39, 48] or insulin [7, 47].

### 3.1. Endothelial Dysfunction in Gestational Diabetes Mellitus

#### 3.1.1. L-Arginine/NO Signalling Pathway

In primary cultures of HUVEC from GDM, synthesis of NO [7, 39, 59], L-arginine transport [39], and its intracellular concentration [16] are increased (Figure 2). GDM-associated increase of L-arginine transport is due to higher maximal velocity (*V*_max⁡_) for transport, most likely resulting from higher hCAT-1 expression [39]. Since general activators of PKC increase L-arginine transport and because activation of p42/44^mapk^ is increased in response to NO and PKC, the mechanisms by which L-arginine transport is activated in GDM in HUVEC seem to depend on these intracellular signalling molecules. 

PKC and p42/44^mapk^ are also involved in the stimulation of L-arginine transport via hCAT-1 by insulin in HUVEC [30, 33, 47, 60]. This phenomenon seems to result from increased *SLC7A1* (for hCAT-1) promoter transcriptional activity via a mechanism involving the zinc finger promoter-selective transcription-factor-specific protein 1 (Sp1) binding to multiple consensus sequences identified between −177 and −105 bp from the ATG (transcription starting sequence) of this gene [33]. Insulin causes relaxation of human umbilical vein rings in an endothelium- and hCAT-like transport activity-dependent manner [33]. Since this vascular response is found using physiological plasma concentrations of insulin (*∼*0.01–0.1 nM), it is feasible that *SLC7A1* expression and most likely hCAT-1 activity are under tonic regulation by physiological insulinemia in human umbilical veins. Insulin-induced umbilical vein relaxation was lower in vessels from GDM compared with normal pregnancies [7]. This phenomenon could be the result of a less reactive umbilical vein, perhaps due to tonic and basally increased vasodilation due to overrelease and/or accumulation of adenosine at the umbilical vein blood [7]. In addition, it is known that insulin effect in patients with insulin resistance is improved by infusion of adenosine receptor agonists suggesting that insulin biological effects could be facilitated upon adenosine receptor activation [61]. This mechanism is also plausible in the human fetoplacental circulation where activation of adenosine receptors is also, apparently, facilitating insulin-increased L-arginine/NO signalling pathway [47]. Altogether these findings could be crucial for fetal insulin modulation of endothelial-derived NO synthesis in human umbilical vessels from pregnancy diseases associated with hyperinsulinemia, such as GDM, and other states of insulin resistance [6, 7, 16, 30, 47].

#### 3.1.2. Adenosine Transport

HUVEC from GDM also exhibit reduced adenosine transport (Figure 3) [6, 16]. GDM effect on adenosine uptake is proposed to result from a lower hENT1 transport capacity (*V*_max⁡_/*K*_m_) due to reduced *V*_max⁡_ rather than altered intrinsic properties (i.e., unaltered apparent *K*_m_) of this type of nucleoside transporters [7, 51, 59]. Since adenosine uptake efficiency (i.e., adenosine molecules per transporter per cell per second) is unaltered in HUVEC from GDM [62], reduced hENT1 expression could explain this effect of GDM. Alternatively, a lower number of nucleoside-binding sites per endothelial cell (~50%) have been estimated in HUVEC from GDM compared with cells from normal pregnancies [62]. In addition, an apparent recycling of hENT1 from the plasma membrane to perinuclear location has been shown in this cell type [63, 64]. Thus, not only a reduced activity and expression but also hENT1 recycling could be a mechanism involved in GDM altered adenosine transport in human fetal endothelium [16, 65, 66]. It is also known that NO inhibits *SLC29A1* (for hENT1) promoter transcriptional activity in HUVEC from GDM, where a higher NO synthesis due to eNOS activation (phosphorylation of eNOS at Ser^1177^ residue) [39] as well as increased total eNOS expression [59] is reported. The *SLC29A1* promoter region spanning from −2154 to −1810 bp from the ATG contains sequence(s) for inhibitory transcription factor(s) leading to downregulation of this gene expression in HUVEC from GDM [59]. Interestingly, GDM effect requires activation of the NO-dependent repressive transcription factors complex conformed by hC/element-binding protein homologous protein 10 (CHOP)-CCAAT/enhancer-binding protein *α* (C/EBP*α*) (hCHOP-C/EBP*α*) [51]. These regulatory mechanisms of hENT1 expression and/or intracellular localization could be key events to understand the recently reported GDM-increased plasma adenosine concentration (~600 nM) in umbilical vein blood [7] compared with normal pregnancies (~350 nM) [7, 67–69]. Reduced expression and/or activity of hENTs is a phenomenon that could also explain the elevated extracellular adenosine concentration detected in the culture medium of HUVEC from GDM (~900–2,000 nM) [7] compared with normal (~50–500 nM) pregnancies [7, 69].

Insulin also reduces hENT1-mediated adenosine transport in HUVEC from normal pregnancies but restores GDM-associated reduced hENT1 expression and activity in this cell type [7, 70]. One of the proposed mechanisms accounting for this beneficial effect of insulin on adenosine transport is an activation of A_2A_-adenosine receptors by extracellular adenosine, which is increased due to reduced hENT1 transport activity in this cell type. In addition, a role for a differential expression of insulin receptor isoforms A (IR-A) and B (IR-B) in HUVEC from GDM is proposed [7]. In this phenomenon insulin would be acting as a factor that restores a potential GDM-associated metabolic phenotype (i.e., preferential activation of p42/44^mapk^ over Akt pathways) to a normal, mitogenic phenotype (i.e., preferential activation of Akt over p42/44^mapk^ pathways) by restoring IR-A expression to values in HUVEC from normal pregnancies [7]. Similar findings have been recently reported for endothelial cells from the microcirculation of the human placenta from GDM pregnancies, where instead a differential role for insulin receptor isoforms is played as modulator of hENT2-mediated adenosine transport [39].

**Figure 4:**
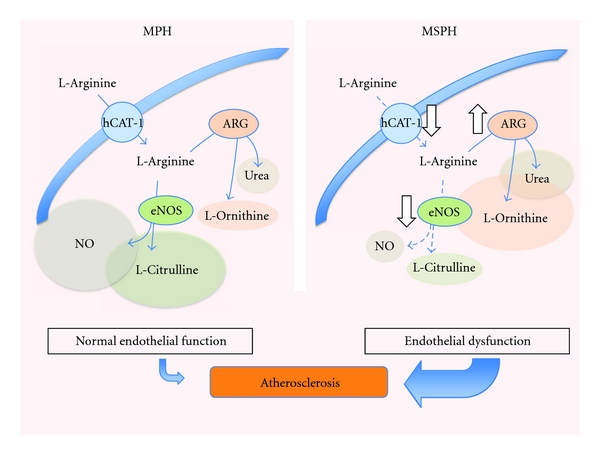
L-Arginine metabolism in hypercholesterolaemia. In human endothelial cells, L-arginine is taken up via cationic amino acid transporter 1 (hCAT-1) which is then metabolized by either the endothelial nitric oxide synthase (eNOS) into L-citrulline and nitric oxide (NO), or via arginases (ARG) into L-ornithine and urea, phenomena conforming a normal endothelial function phenotype. These mechanisms occur in a condition recognized as maternal physiological hypercholesterolaemia (MPH), which has been shown to be associated with early states of fetal vasculature atherosclerosis. However, in a state of maternal supraphysiological hypercholesterolaemia (MSPH) (see text), hCAT-1 and eNOS expression and activity are reduced (white down arrow) leading to reduced (segmented light-blue arrows) L-arginine uptake and NO synthesis, respectively. However, a higher (white up arrow) expression and activity of ARG (most likely arginase 2) leads to increased formation of L-ornithine and urea. The alterations seen in endothelial cells from pregnancies with MSPH result in endothelial dysfunction contributing in a larger proportion to fetal vasculature atherosclerosis compared with MPH. From data in [129, 130, 136, 138].

In a recent study it has been proposed that diabetes mellitus is not triggered in experimental animals where arginases activity is increased, a phenomenon proposed to be due to reduced NO synthesis [71]. These findings highlight the importance of the counterregulatory effect of arginases and NOS in pathologies where vascular tone regulation is altered [72]. It is likely that increased arginase activity leads to lower L-arginine bioavailability for eNOS impairing NO synthesis in the endothelium (see Figure 4). Interestingly, exogenous L-citrulline, but not L-arginine, and inhibition of arginases induce a diabetic phenotype in rats [71]. Therefore, it is also feasible that recycling of L-citrulline to L-arginine could also be involved in this phenomenon. The fact that L-arginine does not induce diabetes could mean that L-arginine availability for NOS is compartmentalized at such degree that it could not reach appropriate concentrations to activate NOS (*K*_m_ of eNOS for L-arginine ranges 1–10 *μ*M) [5], thus limiting the use of this amino acid in the treatment of GDM. What will be the impact of these mechanisms in the fetoplacental circulation, and whether these mechanisms will be associated with programming of adulthood diseases, is unknown.

### 3.2. Dyslipidaemia

GDM is a pathological condition also characterized by maternal dyslipidaemia, alteration directly affecting fetal development and growth [56]. Dyslipidaemia is defined as elevated levels of triglycerides (hypertriglyceridemia) and total blood cholesterol (hypercholesterolemia), including increased low-density lipoprotein (LDL) and reduced high-density lipoprotein (HDL) levels [73]. This phenomenon is associated with the development of endothelial dysfunction and atherosclerosis (a progressive disease characterized by formation of lipid plaques in arteries) [73, 74]. Dyslipidaemia is the main risk factor for development of CVD [73, 75, 76]. Additionally, GDM is a risk factor to fetal programming due apparently to metabolic syndrome [77–79] and, thus, predisposes to an accelerated development of CVD in adult life [78–83]. Interestingly, most of pregnancies with GDM course with dyslipidaemia, thus making feasible a pathological link (i.e., most likely potentiation) between dyslipidaemia in GDM pregnancies and development of CVD later in life. In fact, GDM could play a role in fetal programming of adult CVD not only by alterations in endothelial function of the placenta (mainly triggered by hyperinsulinemia, hyperglycaemia, and changes in nucleoside extracellular concentration) but also by dyslipidaemia associated with this pathology [79, 84].

#### 3.2.1. Hypertriglyceridemia

Pregnancy is a physiological condition characterized by a progressive weeks of gestation-dependent increase (reaching 100–200%) in the maternal blood level of triglycerides [85, 86]. These changes promote accumulation of maternal fat stores in early and mid pregnancy, so to metabolize and use it in late pregnancy. The very-low-density lipoprotein (VLDL) is the type of triglycerides carrier that increases in major proportion in the plasma in hypertriglyceridemia. This phenomenon results from an enhanced VLDL production by the liver and decreased removal of this lipoprotein from the circulation as a consequence of pregnancy-associated hormonal changes, including insulin-resistant condition and elevated plasma oestrogen [85, 87]. The characteristic fetal macrosomia in GDM is also a phenomenon related with alterations in lipid metabolism leading to increased supply of nutrients to the fetus favouring its growth [88]. The association between dyslipidaemia and macrosomia regards hypertriglyceridemia more than hypercholesterolemia; in fact, a positive correlation between maternal triglycerides and neonatal body weight or fat mass has been found in GDM [86, 88, 89]. Furthermore, since triglycerides cross the placenta [1] and contribute to fetal macrosomia [87], maternal plasma concentration of these lipids in the third trimester of gestation, which could result from higher concentration of fatty acids derived from maternal triacylglycerol, is considered as a strong predictor of birth weight in women with GDM [90–92]. This phenomenon is related with altered placenta expression of key proteins involved in *de novo* lipid synthesis (fatty acid synthase and sterol regulatory element-binding protein 2) [93], triglycerides metabolism (placental fatty acid-binding protein) [94, 95], and genes related with placental lipid pathways accounting for placental lipid metabolism and transport (e.g., *PLA2G5* for phospholipase A_2_, *LPL* for lipoprotein lipase, *FACL3* for fatty acid-coenzyme A ligase) [96]. It is accepted that regulation of these genes in GDM alters placenta and fetus lipid metabolism leading to altered fetal development and size, a condition potentiating fetal hyperinsulinemia's biological effects and contributing to the development of the metabolic syndrome and CVD later in life [79, 96].

#### 3.2.2. Hypercholesterolemia

**Figure 5:**
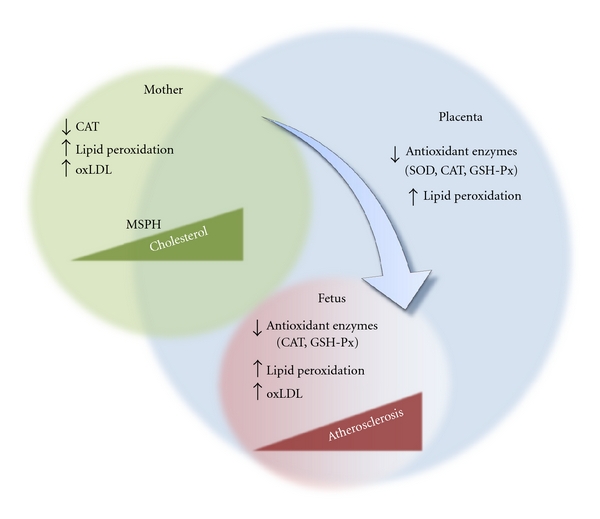
Potential pathophysiological interaction between the mother, the placenta, and the fetus in fetal atherosclerosis. Maternal factors, including reduced (↓) catalase (CAT) activity, increased (↑) lipid peroxidation, and oxidized low density lipoproteins (oxLDL), associated with increased cholesterol content at the mother circulation, generate a state of maternal supraphysiological hypercholesterolaemia (MSPH). This phenomenon leads to similar alterations in the placenta (reduced CAT, superoxide dismutase (SOD), glutathione-peroxidase (GSH-Px) activity) and the fetus (with reduced CAT and GSH-Px and increased lipid peroxidation and oxLDL). Therefore, atherosclerosis in the fetus is identified. Data taken from [88, 100, 101, 109, 110].

Pregnancy is also characterized by a progressive and weeks of gestation-dependent increase (40%–50%) in the maternal blood level of cholesterol [85, 97, 98]. This phenomenon is known as maternal physiological hypercholesterolemia in pregnancy (MPH) and is considered to be an adaptive response of the mother to satisfy the high lipids demand by the growing fetus [85, 86]. However, when a maternal misadaptation to the cholesterol demands by the fetus occurs, a group of these women develop a pathological condition referred to as maternal supraphysiological hypercholesterolemia (MSPH). This condition is characterized by maternal blood cholesterol level to be over the 95th percentile or following the establishment of a cut-point >280 mg/dL [93, 99–101]. Sources of cholesterol for fetal metabolism along with endogenous production by fetal tissues include transplacental mother-*to*-fetus transport of maternal cholesterol [93, 100–106]. Although lipid traffic through the placenta is restrictive, a correlation between maternal and fetal blood cholesterol in the first and second trimesters of pregnancy has been established [100, 107]. These studies suggest that maternal cholesterol level alters normal development of the fetus. In fact, it has been reported that, due to altered lipid metabolism in the placenta as a result of high maternal blood cholesterol, atherogenesis, a clinical complication commonly appearing in adults, probably begins in fetal life with likely similar factors altered in the mother, the fetus, and the placenta (see Figure 5) [100, 108–111]. This phenomenon was for the first time referred to as the “foetal hypothesis of atherosclerosis” [100, 112]. Interestingly, a strong correlation between maternal cholesterolaemia before and during pregnancy and the size of atherosclerotic lesions in arteries of fetus, children, and young adults has been shown [100, 101, 111, 112]. This is apparently crucial regarding fetal programming of CVD [109–113]. Potential clinical implications for this foetal hypothesis of atherosclerosis were further contextualised with the FELIC (“Fate or Early Lesions in Children”) study [101] where the possibility of applying a therapy to mothers with hypercholesterolaemia during pregnancy complemented with described pathogenic insights in the primary prevention of CVD, including stem cell therapy [114], is suggested as a potential way to improve health in their children [101]. Alternatively, C-reactive protein blood levels were described as higher in mothers with hypercholesterolaemia during pregnancy, and this finding was proposed to be used as a predictor of increased atherogenesis in children [115]; however, even when this information is of relevance for preventive medicine, maternal cholesterolaemia seems to be a stronger predictor.

**Figure 6:**
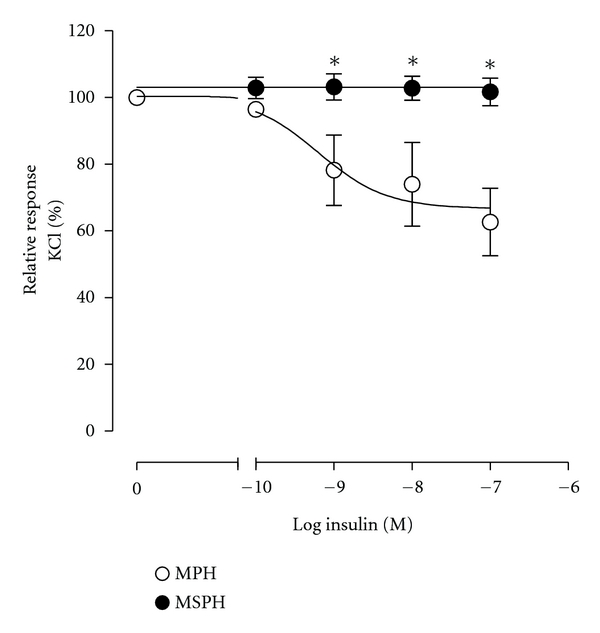
Insulin effect on human umbilical vein ring reactivity. Endothelium-intact human umbilical vein rings were isolated from umbilical veins taken from pregnancies with maternal physiological hypercholesterolaemia (MPH) or maternal supraphysiological hypercholesterolaemia (MSPH). Umbilical vessel ring segments (2–4 mm length) were mounted in a myograph for isometric force measurements with optimal diameter adjusted from maximal active response to 62.5 mM KCl as previously described [28, 107]. Acute response to insulin (3 minutes) was determined in KCl-preconstricted vessels in preparations incubated in Krebs. Values are mean ± SEM (*n* = 7). *P < 0.05 versus corresponding values in MPH.

Placental vascular dysfunction, including altered macro- and microvascular endothelial altered function, is associated with higher risk of developing CVD in adulthood [16, 57]. Cumulative evidence shows that high levels of blood cholesterol modify the endothelial function in different vascular beds [116], mostly associated with reduced vascular NO bioavailability and elevated oxidative stress (Table 1). Unfortunately, nothing is reported regarding whether abnormal maternal blood cholesterol level, including MSPH, leads to placental vascular endothelial dysfunction [109, 117]. GDM correlates with placental macro- and microvascular endothelial dysfunction [16], also considered as early marker of atherosclerosis [77]. Neonates with macrosomia from GDM pregnancies show a significant increase in the aortic intima-media thickness and higher lipid content, both conditions considered as subclinical markers of atherosclerosis [110, 118] and that will potentially increase the atherosclerotic process later in life. Nothing is yet available regarding the potential effect of MSPH in normal or GDM pregnancies regarding development of atherosclerosis in the fetoplacental vasculature in humans [16, 118]. Preliminary findings from our group suggest that MSPH is associated with reduced (in fact almost abolished) vasodilatation of human umbilical vein rings in response to insulin (Figure 6), a phenomenon that could be mediated by endothelial dysfunction since NO synthesis is also altered in HUVEC from these patients [119]. Thus, we speculate that MSPH becomes a pathological condition triggering potentiation of GDM effect on fetal programming of CVD.

 Reduced vascular NO bioavailability and elevated oxidative stress alter vascular reactivity in the placenta [120], as well as in children [121, 122] and adults [120, 123–125], phenomena including downregulation of L-arginine transport and eNOS activity in endothelial cells. Several alterations caused by hypercholesterolemia could explain these changes in vascular reactivity [126]. To date, (a) cholesterol-enriched diet [127] or oxidized low-density lipoproteins (oxLDLs) [128] cause a posttranscriptional downregulation of hCATsmediated L-arginine transport in rat aortic rings and in the human endothelial cell line EAhy926, (b) hypercholesterolemia leads to reduced NOS expression in human saphenous vein endothelial cell, rabbit aortic segments, and HUVEC [129–131], the latter likely due to increased expression of eNOS mRNA destabilizing cytosolic proteins [130, 131], and (c) eNOS cofactor tetrahydrobiopterin (BH_4_) expression is reduced in mice and rabbit aortic rings [132, 133] most likely due to downregulation of guanosine triphosphate cyclohydrolase I (GTPCH, a key enzyme involved in the BH_4_ synthesis) [134, 135]. In addition, hypercholesterolemia is also associated with increased expression and activity of arginases resulting in reduced NO synthesis in human and mice aortic endothelial cells [136–138]. Preliminary results show that in fact arginase II protein abundance is increased in HUVEC from patients with MSPH compared with normal pregnancies (A. Leiva, P. Casanello, and L. Sobrevia, *unpublished results*). Therefore, we speculate that similar mechanisms may be either triggered or potentiated by MSPH with direct consequences in the fetoplacental endothelial L-arginine/NO pathway (Figure 4), a phenomenon not at all evaluated in pregnancies coursing with GDM [16, 86].

## 4. Obesity in Pregnancy

Obesity is a syndrome estimated to be pandemic with a large fraction of children now diagnosed as obese, where causes, other than malnutrition after birth, are not fully explanatory [139]. Obesity is a pathology resulting from a misbalance between the energy intake and energy used, with an overstorage of lipids in adipose tissue [140]. This pathology also courses with systemic metabolic misbalance leading to occurrence of multiple complications, such us dyslipidaemia and insulin resistance [141], and endothelial dysfunction leading to hypertensive disorders (Figure 1) [142, 143]. Incidence of obesity in the world is currently increasing reaching up to ~12% of the population [143]. Worryingly, increased obesity incidence includes *∼*29% of women in their reproductive age [144]. Much evidence now available involves differential contribution of genetic and environmental factors in the development of obesity, diabetes mellitus, or CVD. Thus, prevention of childhood and adult obesity may require beginning even before conception [145–147].

Obesity in pregnancy is associated with fetal mortality and morbidity, congenital malformations, macrosomia, and increased incidence of caesarean delivery [148–151], thus making this syndrome a condition that once declared in pregnancy alters foetal growth and development. Even when an inflammatory profile in placental tissue from obese women has been described [152–154], the consequences of OP on fetoplacental vasculature function, including expression and function of the endothelial L-arginine/NO signalling pathway, remain mostly unknown (Figure 2, Table 1) [16]. Even when GDM [6, 16, 56, 155] and obesity [142, 156, 157] are syndromes associated with altered human vascular function, there are no studies addressing a potential link between placental dysfunction in GDM and OP. However, it is known that OP is associated with higher risk of developing GDM [158], a possibility supported by findings showing that OP correlates with overgrown fetuses [149], intrauterine growth restriction [154], and preeclampsia [159–161]. These results are demonstrative that OP is a key risk factor for pregnancy and fetal development, a condition that could lead to programming of diseases of the adulthood (Figure 1).

### 4.1. Endothelial Dysfunction in Obesity in Pregnancy

Several studies associate obesity with chronic inflammation since blood markers, such as the proinflammatory cytokine interleukin 6 (IL-6) and tumour necrosis factor *α* (TNF*α*), are increased in these patients [162–166]. The endothelium is the first cell line exposed to these cytokines [167–170] leading to altered eNOS expression and activity and reduced NO bioavailability [171–174]. Moreover, placentas from patients with OP exhibit a higher inflammatory profile with increased expression of interleukin 1 (IL-1), IL-8, and chemoattractant protein 1, compared with lean women [153]. These findings are complemented by reports showing obesity-associated increase of IL-6 and TNF*α* level, with higher heterogeneous macrophage infiltration in the human placenta [152]. In addition, in a sheep model of OP describing this inflammatory profile, JNK and NF*κ*B signalling pathway involvement in the placental tissue has been reported [175]. Thus, OP could become a condition altering placental endothelial function with consequences to the fetus at birth and potentially in the adulthood.

Leptin, a hormone whose circulating level is increased in obesity [176], increases system A transport activity through activation of STAT3 and activation of JAK-STAT signalling pathway in human placental villous [177]. However, hyperleptinaemia in obese pregnant women was also shown to correlate with reduced activity of system A, an effect most likely due to increased leptin resistance by the placental tissue [178]. Regarding nucleoside transport, there are no studies addressing this phenomenon, including hENT activity and/or expression, in obese subjects, including pregnant women [142]. Interestingly, NO level is higher in obese subjects [179] and rats [180], and the transcription factor complex hCHOP-C/EBP*α*, known to cause NO-dependent downregulation of *SLC29A1* expression in HUVEC from GDM pregnancies (see above) [51], is also expressed in human adipocytes and involved in the downregulation of expression of other membrane transporters, such as *SCL2A4* (for GLUT4) [181]. In addition, obesity is also associated with altered insulin signalling in several tissues and activates MAPK signalling cascades enhancing insulin resistance [182]. Even when the above-described mechanisms are involved in downregulation of hENT1 expression in the human placental vascular endothelium from GDM, nothing is reported regarding OP effect in this phenomenon.

### 4.2. Postnatal Outcome in Offspring in Obesity in Pregnancy

Prepregnancy obesity and excessive gestational weight gain have been implicated in an intergenerational “vicious cycle” of obesity, since overweight or obese women give birth to macrosomic girls, who are more likely to become obese themselves and deliver large-sized neonates [183]. In fact, gestational weight gain and birth weight were directly associated with the body mass index and the risk of obesity in adolescence [184, 185]. The relationship described was independent of parental characteristics, potentially mediating peripartum factors, child obesogenic behaviour, and weight at birth, suggesting a role of the intrauterine environment on long-term offspring weight regulation. Interestingly, an association between weight gain of the mother during pregnancy and increased risk of greater adiposity in the offspring has been shown at ages of infancy as early as 7 [186] or 3 years old [187]. Considering the high prevalence of OP and its potential association with GDM [158], there is an increasing interest in considering a potentially negative influence of maternal overnutrition and raised birth weight on the risk of disease in childhood and adulthood [148, 183, 188]. Children of obese women exhibiting increased risk of diabetes in pregnancy are more likely to develop insulin resistance later in life [189] (Figure 1). An association between maternal weight gain during pregnancy and pre-pregnancy weight with offspring cardiovascular risk factors in 9 years old children has been proposed (Avon Longitudinal Study of Parents and Children, ALSPAC) [190]. This study shows that women gaining more than recommended weight during gestation were more prone to have offspring with greater body mass index, waist, fat mass, leptin, systolic blood pressure, C-reactive protein, and interleukin-6 levels but lower HDL cholesterol and apolipoprotein A levels than women with a physiological weight gain. Additionally, greater prepregnancy weight was independently associated with greater offspring adiposity and adverse cardiovascular risk factors, agreeing with previous studies [191–195]. Epidemiological studies show that OP increases the incidence of metabolic syndrome in children [188]. Interestingly, OP is related to neonatal metabolic compromise already apparent at birth, characterized by reduced insulin sensitivity and increased serum inflammatory markers [13]. Since OP effect on the susceptibility to obesity in offspring is apparently independent of GDM, as obese women with normal blood glucose have babies with increased adiposity [196], OP and excessive maternal weight gain during pregnancy are independent factors leading to increased risk of obesity, insulin resistance, and early markers of CVD in the offspring. All this evidence shifts our attention towards the gestational period as an extremely key interventional target in the prevention of obesity and associated consequences such as insulin resistance and cardiovascular risk.

#### 4.2.1. Mechanisms of Adverse Postnatal Outcome

The molecular mediators and signalling pathways from the mother to program the metabolic phenotype (i.e., obesity and insulin resistance) of the developing offspring are not fully elucidated. Hormones, such as leptin and insulin, or nutrients, such as D-glucose, free fatty acids, and triglycerides, and multiple inflammatory cytokines could be implicated. During normal intrauterine life, maternal insulin does not cross the placenta, whereas maternal D-glucose is actively transferred to the fetus [197]. The developing fetal pancreas responds to a D-glucose load by increasing synthesis and release of insulin, which acts as a fetal growth hormone. This is the basic concept of the “Pedersen's hyperglycaemia-hyperinsulinism hypothesis” (where fetal overgrowth due to hyperinsulinemia in response to increased transplacental D-glucose transfer is proposed, as recently reviewed [224]) explaining observations showing that offspring of diabetic mothers exhibit high birth weight [225]. Further analysis expanded this theory to include the possibility that other insulin secretagogues, including free fatty acids, ketone bodies, and amino acids [197]. Maternal overnutrition produces hyperglycaemia, which leads to increased fetal insulin secretion in a similar manner as seen in GDM [226]. Thus, secondary fetal hyperinsulinemia is believed to be involved in the intrauterine programming of obesity and diabetes [188]. Prospective studies indicate that at birth and at 6 years old the greatest increase in weight to height relation (relative obesity) was seen in children who experienced the greatest exposures to insulin in uterus (as judged by amniotic fluid insulin concentration) [197]. 

Leptin is also implicated in programming obesity. In humans, leptin is increased in OP and maternal diabetes and is reduced in intrauterine growth restriction [227]. Although the placental transfer of leptin has been demonstrated *in vivo* [228], it is believed that umbilical blood level of this circulating peptide is a marker of neonatal adiposity more than a relevant modulator of fetal growth [227]. Additionally, several inflammatory cytokines levels are elevated in obese pregnant women [229], changes that are proposed as potential mediators of metabolic programming. Thus, altered metabolic phenotypes, such as obesity and insulin resistance seen in offspring in OP, could partially be explained by the involvement of multiple mediators. Probably, a multifactorial contribution of nutrient- (e.g., D-glucose, fatty acids, amino acids) and hormone- (e.g., insulin, leptin) triggered signals between the obese mother and the developing fetus would better describe the involved mechanisms. Recent studies suggest a strict metabolic control of the mother with GDM in order to overcome the adverse effects of this pathology on the fetal outcome [46, 230–232]. However, adverse effects of GDM environment on fetal tissues persist in time, and multiple studies show increased risk to develop metabolic syndrome in offspring of GDM pregnancies [70, 169, 192]. More recently it was shown that individuals born from GDM pregnancies are prone to develop obesity and D-glucose intolerance compared with offspring from normal pregnancies [198, 199]. However, further research is needed to understand the specific mechanisms of metabolic programming in response to altered intrauterine environment derived from OP and GDM.

## 5. Concluding Remarks

Fetoplacental endothelial dysfunction is a common characteristic of several diseases in pregnancy limiting the function of the placenta vasculature leading to altered fetal growth and development. These phenomena involve altered capacity of one of the essential functions of the endothelium, that is, the synthesis of vasoactive molecules, including NO. It is now established that GDM and OP are pathological conditions altering hCAT-mediated L-arginine transport and eNOS-synthesis of NO (i.e., the “endothelial L-arginine/NO signalling pathway”) in the human fetoplacental vasculature. This phenomenon results in abnormal function of the endothelial L-arginine/NO signalling pathway leading to altered vascular reactivity and changes in umbilical vessels blood flow from and to the fetus with serious consequences on its growth. Abnormalities in the endothelial L-arginine/NO signalling pathway are also dependent of several regulatory mechanisms, including up-regulation caused by activation of A_2A_-adenosine receptors in the micro- and macrovasculature of the human placenta in GDM (and perhaps in OP) due to accumulation of extracellular adenosine resulting from reduced hENT expression and activity. Interestingly, GDM pregnancies course with dyslipidaemia (hypertriglyceridemia and hypercholesterolemia) and a pathological link between this condition and development of CVD later in life is likely. A proper management of GDM and OP would be of benefit for the actual newborn's health condition and is crucial for the developing of diseases in the adulthood. Altered function of fetal endothelium at birth is a “metabolic altered state” associated with GDM and OP. We hypothesize that this phenomenon is a potential characteristic (or “*at birth* metabolic marker”) that could be considered as predictor of diseases of the adulthood (e.g., CVD, obesity, diabetes mellitus, metabolic syndrome) resulting from a programmed state due to diseases of pregnancy.

##  Conflict of Interests

Authors declare that they have no conflict of interest.

## Figures and Tables

**Table 1:** Effect of GDM, obesity, and hypercholesterolaemia on ALANO signalling pathway.

Element	Pregnancy			Nonpregnancy					
	GDM			Obesity			Hypercholesterolemia		
	Cell type	Effect	References	Cell type	Effect	References	Cell type	Effect	References
hENT1 expression	HUVEC	Reduced	[7, 39, 51]						
	hPMEC	Reduced	[16, 44]						
hENT1 activity	HUVEC	Reduced	[7, 39, 51]						
	hPMEC	Reduced	[16, 44]						
hENT2 expression	HUVEC	Unaltered	[16]						
	hPMEC	Reduced	[16, 44]						
hENT2 activity	HUVEC	Unaltered	[16]						
	hPMEC	Reduced	[16, 44]						
Extracellular adenosine	HUVEC	Increased	[7, 48]						
hCATs, expression	HUVEC	Increased	[39]	hP	Reduced	[213]	EAhy926	Increased	[128]
							rAR	Increased	[127]
hCATs, activity	HUVEC	Increased	[39]	hP	Reduced	[213]	EAhy926	Increased	[128]
							rAR	Reduced	[127]
							bAEC	Reduced	[214]
							pAEC	Reduced	[215]
							HUVEC	Unaltered	[216]
							HUVEC	Unaltered	[217]
eNOS expression	HUVEC	Increased	[39, 51]	hVEC	Unaltered	[173]	hSVEC	Reduced	[129]
	hPT	Increased	[218]	mVEC	Increased	[219]	rbAS	Reduced	[131]
				hAd	Increased	[220]	HUVEC	Reduced	[130]
				hHep	Unaltered	[221]			
eNOS activity	HUVEC	Increased	[7, 39, 51]	hVEC	Reduced	[173]	hSVEC	Reduced	[129]
	hVT	Unaltered	[222]	mVEC	Reduced	[219]	rbAR	Reduced	[131]
				mHep	Reduced	[223]	HUVEC	Reduced	[130]
				hP	Unaltered	[213]	pAEC	Reduced	[215]
NO level	HUVEC	Increased	[11]	*	Increased	[221]	hSVEC	Reduced	[129]
Arginase 1				mHep	Increased	[223]			
Arginase 2							hAEC	Increased	[136–138]
							mAEC	Increased	[137, 138]

hENT1: human equilibrative nucleoside transporter 1; hENT2: human equilibrative nucleoside transporter 2; hCATs: human cationic amino acid transporters; eNOS: endothelial nitric oxide synthase; NO: nitric oxide; HUVEC: human umbilical vein endothelial cell; hPMEC: human placental microvascular endothelial cell; hPT: human placental tissue; hVT: human villous tissue; hP: human platelets; hVEC: human vascular endothelial cell; mVEC: mouse vascular endothelial cell; hAd: human adipocyte; hHep: human hepatocyte; mHep: mouse hepatocyte; EAhy 926: human endothelial cell line EAhy 926; rAR: rat aortic ring; bAEC: bovine aortic endothelial cell; pAEC: porcine aortic endothelial cell; hSVEC: human saphenous vein endothelial cell; rbAS: rabbit aortic segment; rbAR: rabbit aortic ring; hAEC: human aortic endothelial cell; mAEC: mouse aortic endothelial cell; *measurement performed in human serum.
